# Design of a Current Sensing System with TIA Gain of 160 dBΩ and Input-Referred Noise of 1.8 pA_rms_ for Biosensor

**DOI:** 10.3390/s23063019

**Published:** 2023-03-10

**Authors:** Donggyu Kim, Sungjun Byun, Younggun Pu, Hyungki Huh, Yeonjae Jung, Seokkee Kim, Kang-Yoon Lee

**Affiliations:** 1 Department of Electrical and Computer Engineering, Sungkyunkwan University, Suwon 16419, Republic of Korea; 2 SKAIChips Co., Ltd., Suwon 16419, Republic of Korea

**Keywords:** biosensor, current sensing, calibration, ASIC

## Abstract

This paper proposes a high-gain low-noise current signal detection system for biosensors. When the biomaterial is attached to the biosensor, the current flowing through the bias voltage is changed so that the biomaterial can be sensed. A resistive feedback transimpedance amplifier (TIA) is used for the biosensor requiring a bias voltage. Current changes in the biosensor can be checked by plotting the current value of the biosensor in real time on the self-made graphical user interface (GUI). Even if the bias voltage changes, the input voltage of the analog to digital converter (ADC) does not change, so it is designed to plot the current of the biosensor accurately and stably. In particular, for multi-biosensors with an array structure, a method of automatically calibrating the current between biosensors by controlling the gate bias voltage of the biosensors is proposed. Input-referred noise is reduced using a high-gain TIA and chopper technique. The proposed circuit achieves 1.8 pA_rms_ input-referred noise with a gain of 160 dBΩ and is implemented in a TSMC 130 nm CMOS process. The chip area is 2.3 mm^2^, and the power consumption of the current sensing system is 12 mW.

## 1. Introduction

Application-specific integrated circuits (ASICs) for biosensor applications are often used when sensing current or voltage signals. Biosensors used in biomedical applications range from microneedle-based [1], film-based [2], to nanopore-based DNA analysis [3,4], and usually require sub-nanoampere current sensing. In particular, nanopores are widely used both in DNA analysis and in biomolecule detection applications such as amino acid analysis [5,6], ion analysis [7], protein and peptide analysis [8,9,10,11], and the detection of specific viruses using antibody–virus interactions [12]. Using this method of detecting specific biomolecules, it is possible to determine whether a person is infected with a specific virus, or has a disease, such as cancer [13,14]. The nanopore can control the current with the gate voltage, or be turned off so that the current does not flow [15,16,17]. In addition, since nanopores can be fabricated as an array [18,19], each nanopore can react to a different virus; and by controlling each nanopore with a gate voltage and reading each current, multiple viruses can be detected with a single biosensor.

In applications where the output current of the biosensor is very small, the use of a high-gain TIA at the front end of the ASIC is used. The TIA is mainly used with resistive feedback or capacitive feedback topologies. In a resistive feedback configuration, the resistor must be very large for low input-referred noise and high gain. To prevent the large resistance from increasing the circuit area, a pseudo-resistive structure using an active element as a feedback network to achieve a large resistance effect is widely used [20,21]. However, this approach cannot be used in applications where the TIA’s input voltage may change, because the resistance value of the pseudo-resistor changes with the DC voltage across it. The capacitive feedback architecture [22] can obtain a large gain using a relatively small area, but it cannot be used for biosensors that output DC input current because it can receive only AC input current. Additionally, since the biosensor’s output current changes depending on the DC voltage bias, it cannot be used for biosensors that require an accurate DC voltage bias.

**Figure 1:**
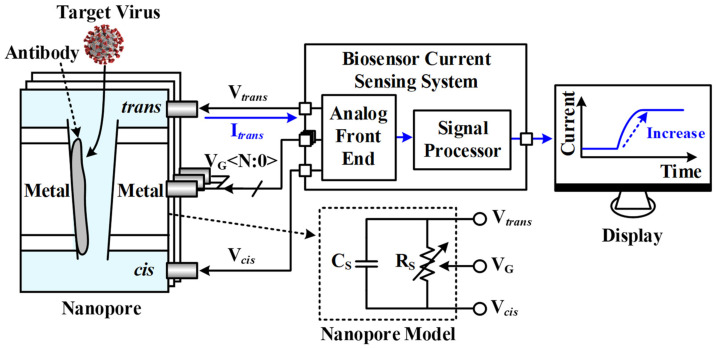
Nanopore-based virus sensing system.

A nanopore-based virus detection application, as shown in Figure 1, contains an ionic solution to which a DC voltage is applied to the nanopore array. The target virus enters the nanopore and combines with the antibody attached to the nanopore, resulting in a change in current, while the non-target virus cannot bind to the receptor and escapes, leaving no change in current. The current change at this time varies from nanoampere to microampere range depending on the bias voltage, so the gain control of the TIA is required, and a low-noise and high-gain TIA is required to accurately distinguish the signal at the nanoampere level. The proposed current sensing system converts the current signal into digital data and plots it in real time to check the current change caused by the target virus.

The focus of this paper is a high-gain low-noise current sensing and display system. The ADC’s range, offset, and TIA gain values are applied to the digitized input current value to convert it into an actual current value, and the input current is plotted as a display on a portable device or laptop. Resistive feedback is used to supply DC voltage to the biosensor, and a passive resistor is used instead of an active resistor, whose gain varies with the voltage across it. In addition, a circuit that can stably plot the current even when the biosensor’s bias voltage changed is designed, and a chopper technique is used to reduce noise.

The rest of the paper is organized as follows: Section 2 presents the proposed current sensing and display system, Section 3 presents the measurement results of the fabricated ASIC, and Section 4 summarizes the main conclusions.

## 2. The Proposed Current Sensing System

**Figure 2:**
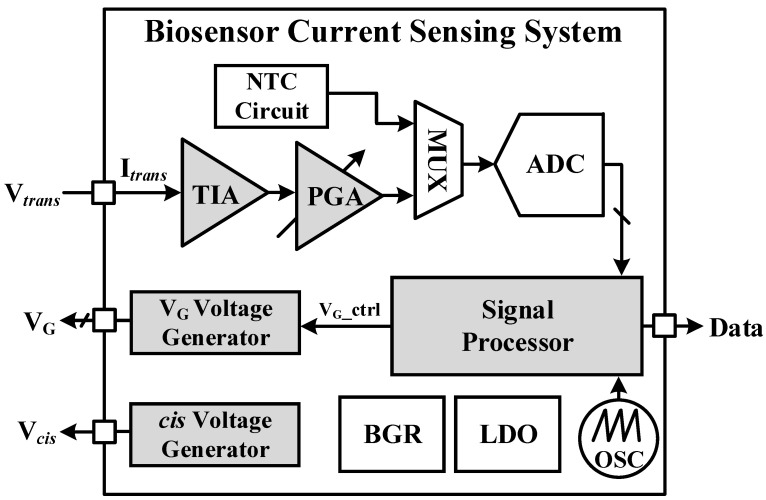
Block diagram of the proposed current sensing system for biosensor.

Figure 2 shows a block diagram of the proposed current sensing system for biosensor. The main building blocks of the proposed biosensor current sensing system IC are a TIA, a programmable-gain amplifier (PGA), an ADC, a voltage generator, a negative temperature coefficient (NTC) sensor, a bandgap voltage reference (BGR), low-dropout (LDO), an oscillator (OSC), and signal processor. The TIA converts the biosensor’s output current into a voltage. The biosensor requires bias voltage to be supplied to the *trans* for current to flow, so the TIA is designed to supply bias voltage and sense current at the same time. The PGA amplifies the signal converted to voltage, and sets the offset voltage to match the input range of the ADC. The ADC consists of a sigma delta modulator architecture and a cascaded integrator-comb digital filter to provide a high resolution. NTC sensors can measure temperature as the voltage decreases and as the temperature increases. The area is reduced by using a MUX to allow the selection of PGA output or NTC with one ADC. The BGR and LDO supply voltage stably and the OSC generates clock. The signal processor controls the building blocks, and sends the current and temperature data of the biosensor to the external environment through serial peripheral interface (SPI) communication. The current flow can be controlled by adjusting the gate bias voltage (V_G_) and *cis* voltage of the biosensor with the voltage generator. In particular, by adjusting the gate bias voltage, the basic current that is initially different for each biosensor is calibrated so that the same current flows.

**Figure 3:**
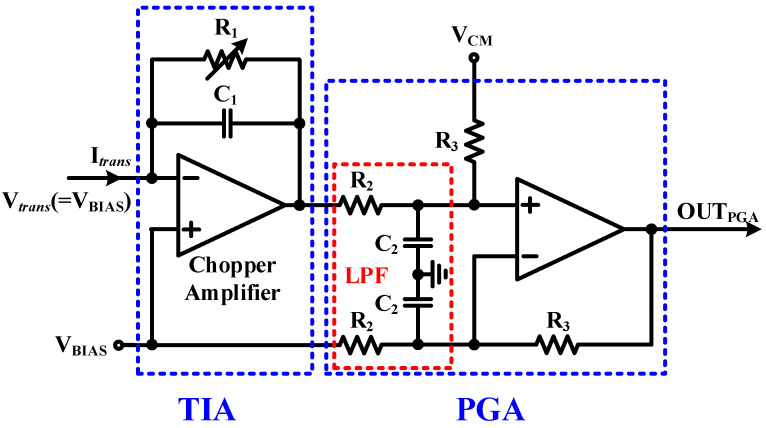
Schematic of the TIA and PGA.

Figure 3 shows the proposed high-gain low-noise TIA and PGA. The TIA converts current signal into voltage with high gain, and the PGA performs fine gain control and output voltage level control. The TIA’s gain, R_1_, requires a very large resistor for low input-referred noise. To supply voltage to the biosensor, resistive feedback is used instead of capacitive feedback, and a real resistor is used instead of a pseudo-resistor to maintain a constant gain, even when the V*_trans_* voltage changes. To reduce noise, a C_1_ capacitor is added in parallel to the R_1_ resistor to prevent high-frequency components from being amplified. The PGA uses the TIA output and the V_BIAS_ voltage as inputs, and the output offset level can be adjusted with the V_CM_ voltage. While R_2_ and R_3_ are usually the same, a variable resistor can be used to fine-tune the gain. The equation from the input of the TIA to the output of the PGA is described as

OUT_PGA_ = V_CM_ − R_1_ × I*_trans_* × R_3_/R_2_ (1)

As can be seen from Equation (1), adjusting V_BIAS_ does not affect the output voltage of the PGA. The chopper circuit needs a low-pass filter (LPF) to remove the chopper frequency component, and the LPF is configured by adding a C_2_ capacitor to the R_2_ resistor of the PGA. Table 1 shows the passive element sizes and the biasing voltage values of the proposed TIA and PGA.

**Figure 4:**
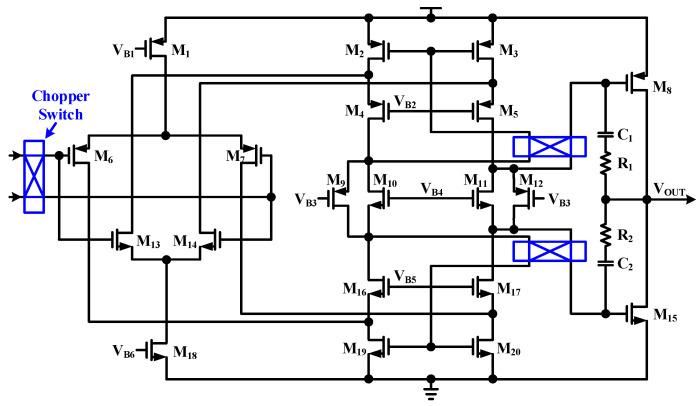
Schematic of the chopper amplifier used in the TIA.

Figure 4 shows the chopper amplifier circuit used in the TIA. The input range is wide and the gain is high by using a rail-to-rail folded cascade structure [23]. The second stage consists of class AB buffers and M_9_, M_10_, M_11_ and M_12_ are used as floating current sources for the bias of class AB buffers. C_1_, C_2_, R_1_, and R_2_ are used for frequency compensation. There is a switching circuit that can change the phase in two places: the first stage input and the second stage input. If all switching circuits are switched at the same frequency, even if the phase is changed by 180 degrees in one switching circuit, the original phase is returned to the output of the amplifier through the two switching circuits. The offset caused by the mismatch of the elements passes through the switching circuit once to the output of the amplifier, and the phase of the offset changes 180 degrees each time it is switched, and appears as a pulse at the output of the amplifier. The offset of the amplifier modulated by the switching frequency can be removed by using the LPF after the output of the amplifier. The switching frequency must be higher than the frequency of the signal to keep the biosensor signal as it is. The implemented transistor sizes and biasing voltage value of the proposed chopper amplifier are shown in Table 2.

**Figure 5:**
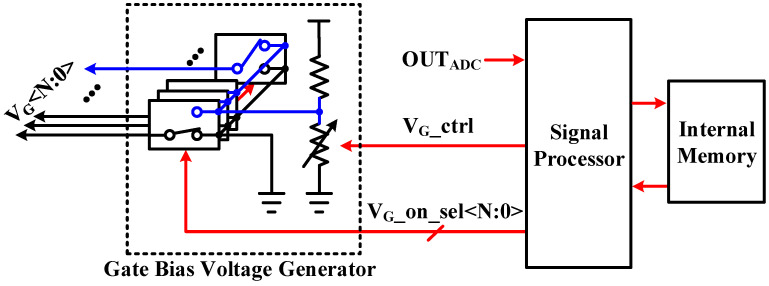
Schematic of the gate bias voltage generator circuit.

Figure 5 shows the gate bias voltage generator circuit that provides the gate bias voltage to the biosensor. A biosensor is composed of several nanopores, and by using the fact that current flows only through nanopores with a high gate bias voltage among each, only one nanopore can be selected to allow current to flow. In addition, it is possible to control the current flowing through the biosensor by adjusting the gate bias voltage. Only one of the N single pole double throws (SPDT) is connected to the high voltage, corresponding to on, and the others are connected to the low voltage, corresponding to off. V_G__ctrl, which controls the resistance, is 6 bits, and the voltage corresponding to on can be adjusted in 50 mV steps. After current calibration of the biosensor is performed, the calibrated V_G__ctrl value for each selected nanopore is stored in the internal memory, and this value is called during actual measurement so that all nanopores have the same initial current.

**Figure 6:**
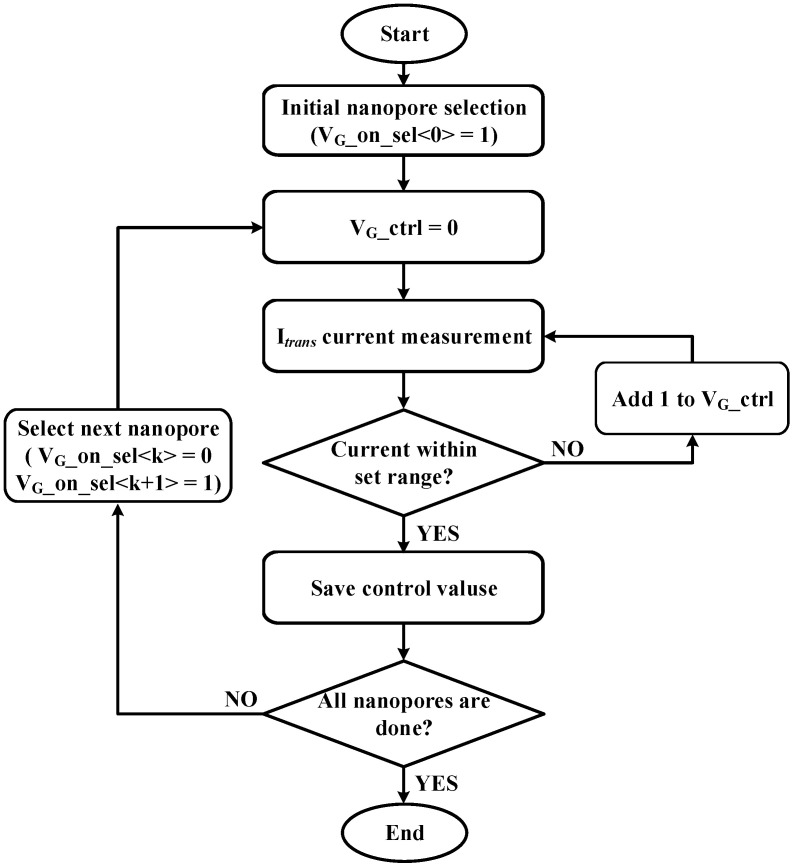
Current mismatch calibration flow chart.

When nanopores are manufactured, the depths or diameters of the holes are not the same, so the initial current flowing through the holes of each nanopore may differ. If the initial current is different, the current change by the target virus is also different, which may cause errors in discrimination. To prevent this, the gate bias voltage is adjusted to calibrate so that the initial current flowing through all the nanopores is the same. Figure 6 shows the flowchart of current mismatch calibration. After selecting a nanopore by turning on the gate bias voltage of one nanopore, the gate bias voltage control bit (V_G__ctrl) starts from 0, checks the current value, and increases it one by one. When the current value is within the set target range, the gate bias voltage control bit at that time is saved. After selecting the next nanopore, the process is repeated to save the voltage control bit. When the calibration is finished, the gate bias voltage control bit that allows the current to flow at the set value for each nanopore is stored. Afterwards, in the actual measurement for virus discrimination, when each nanopore is selected, the automatically stored gate bias control bit is loaded and the initial current flowing is the same.

## 3. Experimental Results

**Figure 7:**
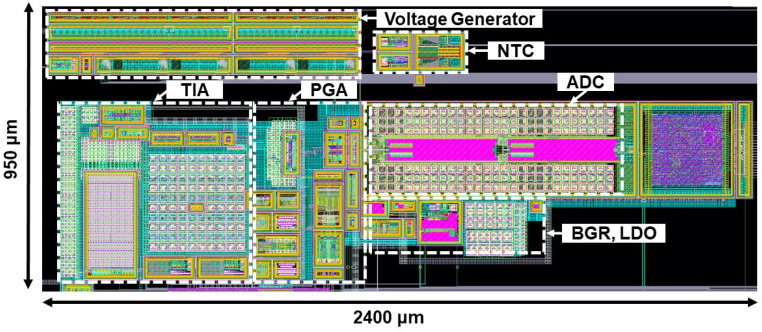
Layout pattern of the proposed current sensing system.

The proposed current sensing system was fabricated in a TSMC 130 nm CMOS process. Figure 7 shows the layout of the ASIC, where the total area can be seen to be is 2.4 mm × 0.95 mm.

**Figure 8:**
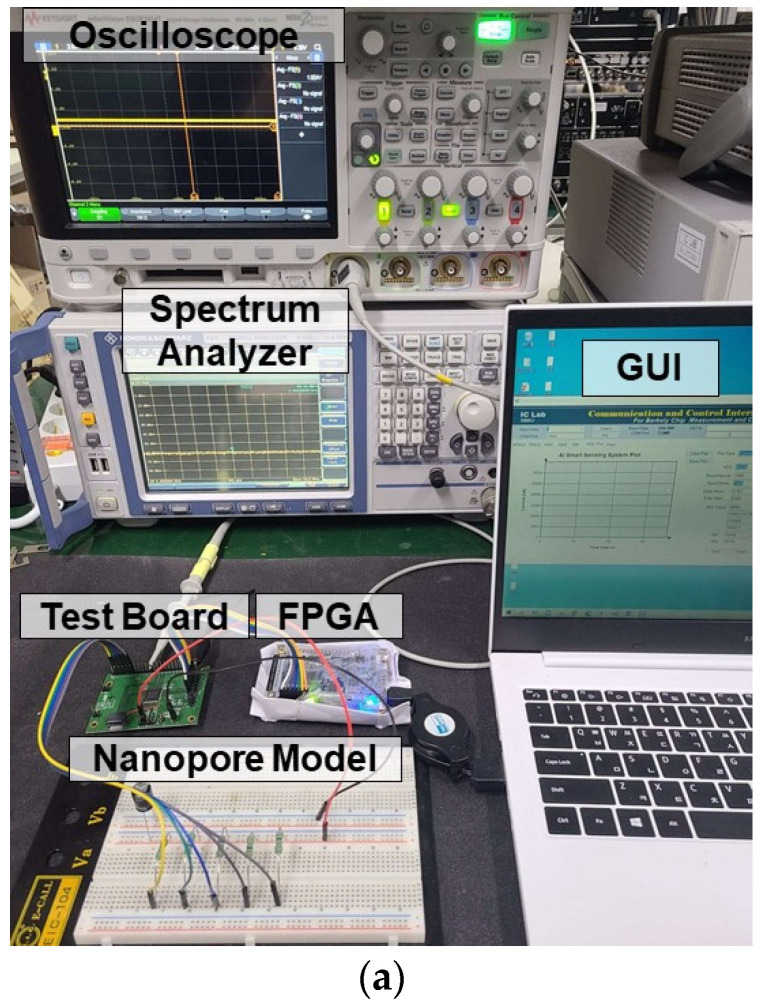

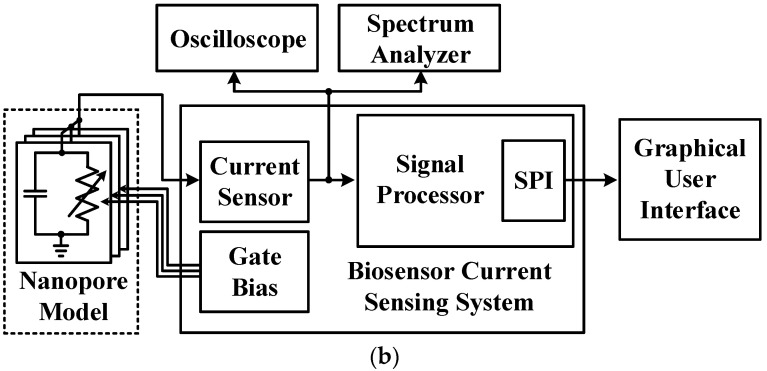
Measurement setup. (**a**) Measurement environment. (**b**) Measurement setup block diagram.

Figure 8 shows the measurement setup to measure the proposed current sensing system design after fabrication. Figure 8a shows the actual test lab environment, while Figure 8b is a block diagram of the measurement environment. To measure the current sensing system, the nanopore model was composed of a resistor and a capacitor so that the current changed with the applied voltage. The current signal input to the current sensing system was converted into voltage, digitized, processed, and transmitted to the computer using SPI communication. The data transmitted to the computer could be plotted in real time through the self-made GUI. The signal converted to voltage was input to the oscilloscope and spectrum analyzer through the test port to measure the performance of the current sensing system.

**Figure 9:**
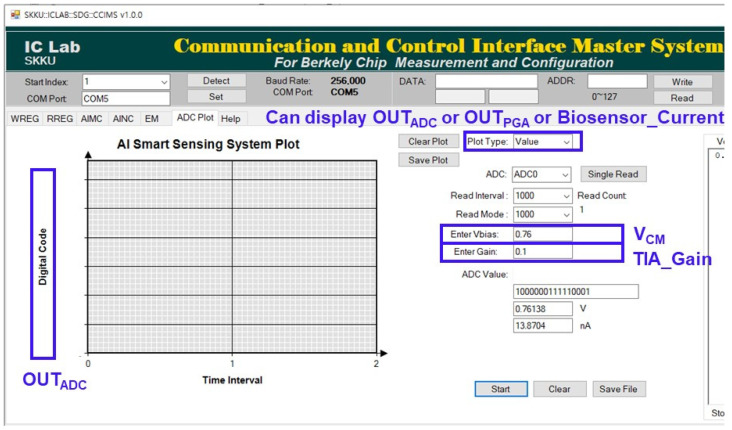
Display screen of the self-made GUI.

Figure 9 shows the GUI that receives the output data of the current sensing system and plots it in real time. The screen can be plotted by selecting among the ADC output digital data, PGA output voltage, and biosensor output current. The *X*-axis is time, while the *Y*-axis is the data converted from ADC output digital data to decimal or PGA output voltage or biosensor output current. The PGA output voltage and biosensor output current can be calculated with ADC output digital data using the designed TIA gain and PGA output common-mode voltage level (V_CM_). The input range of the designed ADC was from 0 V to the supply voltage. The formula is:

OUT_PGA_ = OUT_ADC_/2^ADC_Resolution^ × ADC_Input_Range (2)

Biosensor_Current = (OUT_PGA_ − V_CM_)/TIA_Gain (3)

**Figure 10:**
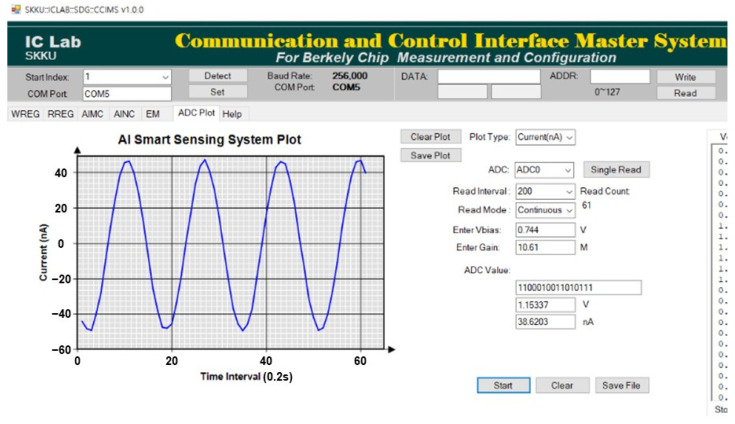
Measured result of the current plot function in the GUI.

Figure 10 shows the result of measuring the current plot function of the GUI. When 100 nA_pp_, a 0.3 Hz sine wave current was applied to the input of the current sensing system, and the same amplitude and frequency as the input current were plotted on the GUI in real time.

**Figure 11:**
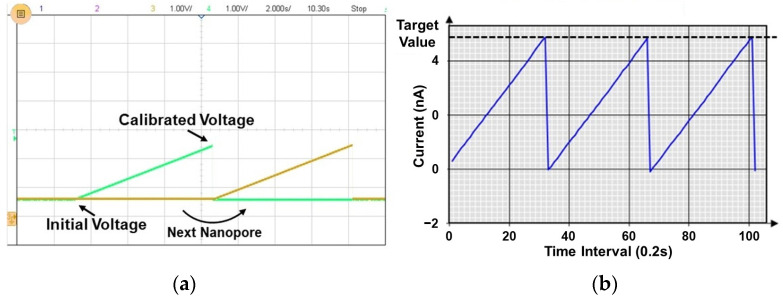
Measured result of current mismatch calibration. (**a**) Gate bias voltage. (**b**) OUT_ADC_ data.

Figure 11 is the measurement result of current mismatch calibration. Figure 11a shows that the voltage is increased by increasing the gate bias voltage control bit of the selected nanopore from 0 with the other nanopores turned off. As shown in Figure 11b, when the nanopore current reached the target current value, the gate bias voltage control bit at this time was saved and the next nanopore was selected. The same operation was performed for all nanopores to store the calibrated gate bias voltage control bit. When the calibration was finished and the actual measurement was performed, the gate control bit of each saved nanopore was loaded to make the base current of all nanopores the same.

**Figure 12:**
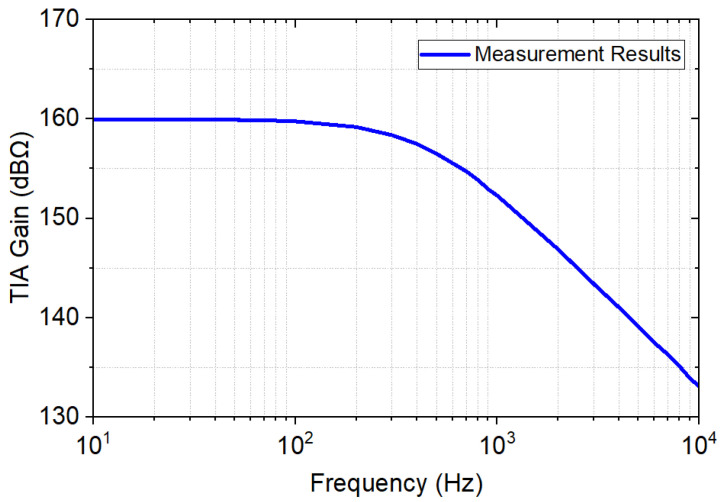
Measured result of the transimpedance-gain frequency response of the TIA.

Figure 12 shows the measured transimpedance-gain frequency response of the TIA. The TIA’s in-band gain was 160 dBΩ and its bandwidth was 470 Hz.

**Figure 13:**
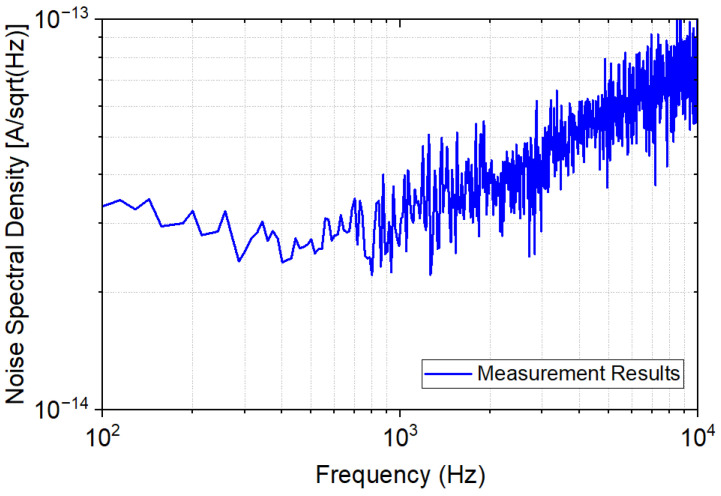
Measured result of input-referred noise.

Figure 13 shows the measured input-referred noise performance of the TIA. The input-referred noise of 1.8 pA_rms_ was confirmed by calculating the root mean square value in the frequency range from 100 Hz to 10 kHz.

Table 3 shows the comparison summary of the proposed current sensing system for the biosensor. The proposed current sensing system showed a similar input-referred noise performance to other designs using capacitive feedback topologies. The power consumed by the entire current sensing system is 12 mW, of which the TIA consumes 0.4 mW.

## 4. Conclusions

This paper proposes a biosensor current sensing system that can amplify the output signal of a biosensor that outputs a current signal, especially a nanopore sensor, and convert it into digital data to plot and analyze it in real time. The proposed biosensor current sensing system used resistive feedback for biosensor voltage bias. Digitized current signal data, which is the ADC output, can be displayed as the current value of the biosensor using the designed TIA gain and PGA output common-mode voltage level. The biosensor current sensing system proposed in this paper has a 160 dBΩ gain and 1.8 pA_rms_ of input-referred noise in the frequency range from 100 Hz to 10 kHz. The power consumption of the proposed current sensing system is 12 mW.

## Figures and Tables

**Table 1:** Passive element sizes and biasing voltage value of the proposed TIA and PGA.

Components	Parameter
R_1_	1 kΩ~100 MΩ
R_2_	100 kΩ
R_3_	100 kΩ
C_1_	10 pF
C_2_	100 pF
V_CM_	0.75 V

**Table 2:** Transistor sizes and biasing voltage value of the proposed chopper amplifier.

Components	Value	Components	Value
M_1_, M_2_, M_3_	48 µm/3 µm	M_18_, M_19_, M_20_	16 µm/3 µm
M_4_, M_5_, M_6_, M_7_	24 µm/3 µm	V_B1_	3 V
M_8_	48 µm/3 µm	V_B2_	2.6 V
M_9_, M_12_	12 µm/3 µm	V_B3_	1.4 V
M_10_, M_11_	4 µm/3 µm	V_B4_	2.7 V
M_13_, M_14_, M_16_, M_17_	8 µm/3 µm	V_B5_	1.6 V
M_15_	16 µm/3 µm	V_B6_	1.2 V

**Table 3:** Performance summary and comparison table.

Parameter	[22]	[24]	[25]	This Work
Technology (nm)	130 CMOS	350 CMOS	130 CMOS	130 CMOS
Feedback topology	capacitive	resistive	capacitive	resistive
Active area of TIA (mm^2^)	0.2	0.3	0.2	0.36
Power consumption of TIA (mW)	30	0.5	5	0.4
TIA Gain (dBΩ)	170	168	160	160
Input-referred noise (pA_rms_)	0.4	4.2	1	1.8

## Data Availability

Not applicable.
